# Corticospinal interaction during isometric compensation for modulated forces with different frequencies

**DOI:** 10.1186/1471-2202-11-157

**Published:** 2010-12-31

**Authors:** José R Naranjo, Xi Wang, Jürgen Schulte-Mönting, Frank Huethe, Christoph Maurer, Marie-Claude Hepp-Reymond, Rumyana Kristeva

**Affiliations:** 1 Neurological Clinic, University Freiburg, Breisacherstraße 64, 79106 Freiburg, Germany; 2 Institute for Medical Biometry and Medical Informatics, Freiburg, Germany; 3 Institute of Neuroinformatics, University of Zürich and ETH Zürich, Switzerland

## Abstract

**Background:**

During isometric compensation of modulated low-level forces corticomuscular coherence (CMC) has been shown to occur in high-beta or gamma-range. The influence of the frequency of force modulation on CMC has up to now remained unexplored. We addressed this question by investigating CMC, motor performance, and cortical spectral power during a visuomotor task in which subjects had to compensate a modulated force of 8% of the maximum voluntary contraction exerted on their right index finger. The effect of three frequencies of force modulation (0.6, 1.0 and 1.6 Hz) was tested. EEG, EMG from first dorsal interosseus, hand flexor and extensor muscles, and finger position were recorded in eight right-handed women.

**Results:**

Five subjects showed CMC in gamma- (28-45 Hz) and three in beta-range (15-30 Hz). Beta- and gamma-range CMC and cortical motor spectral power were not modulated by the various frequencies. However, a sharp bilateral CMC peak at 1.6 Hz was observed, but only in the five gamma-range CMC subjects. The performance error increased linearly with the frequency.

**Conclusions:**

Our findings suggest that the frequency of force modulation has no effect on the beta- and gamma-range CMC during isometric compensation for modulated forces at 8% MVC. The beta- and gamma-range CMC may be related to interindividual differences and possibly to strategy differences.

## Background

In the last two decades, corticomuscular synchronization during isometric compensation of static forces has been extensively studied. Beta-range (15-30 Hz) corticomuscular coherence (CMC) has been reported between motor cortical neurons and muscles in monkeys [1-4] and between sensorimotor cortex and muscle activity (EMG) in humans [5-14].

Several investigations focused on the mechanisms by which cortex drives the muscles under dynamic conditions. It was shown that gamma-range (above 30 Hz) CMC reflected effective corticospinal interactions and different gamma-subranges were associated with various motor tasks [15-19]. For instance, Schoffelen et al [15] found that the subjects' readiness to respond in a simple reaction-time task was closely correlated with the strength of one gamma-range (40-70 Hz) CMC between motor cortex and EMG activity. In addition, Brown and colleagues [20] showed that, while weak static forces were accompanied by beta-range CMC, gamma-range (35-60 Hz) CMC occurred mainly for submaximal and maximal forces. Significant ECoG-EMG coherence in the high gamma subrange (61-100 Hz) was also reported during phasic movements [21]. For a visuomotor isometric compensation of a periodically modulated force at 4% MVC we found a lower (30-45 Hz) CMC gamma-subrange [18,22,23]. We suggested that this low gamma-subrange reflects the rapid integration of proprioceptive, visual and cognitive (preparatory attention) information required to produce the appropriate motor command. Since we had found that this gamma-range CMC is not modulated by the amplitude of the modulated force [23] we wondered whether it would be modulated by various frequencies.

We addressed this question investigating the CMC, cortical motor spectral power, as well as motor performance during a visuomotor task, where subjects compensated isometrically a periodically modulated force at 8% MVC with three frequencies (0.6, 1.0 and 1.6 Hz).

We tested the following predictions:

**First**, based on our earlier results showing a shift of the CMC from beta- to gamma-range during compensation of static and periodically modulated force respectively [18,23,24], we predicted that with increase in frequency modulation the CMC will be shifted towards higher frequencies in order to effectively integrate sensorimotor information.

**Second**, recent studies on corticospinal interaction during rhythmic hand movements have reported that increased beta-range CMC was accompanied by a CMC peak at the frequency of the movement or of the periodic muscle contraction [25,26]. Based on these results, we predicted CMC peaks at the frequencies of modulated force. We also speculated that these low-frequency CMC peaks should be stronger for more difficult tasks and hence, for higher frequencies.

We found that beta- and gamma-range CMC and cortical motor spectral power were not modulated by the various frequencies of the modulated force. However, a sharp bilateral CMC peak at 1.6 Hz was observed, but only in the five gamma-range CMC subjects. Our findings suggest that the tested frequency of force modulation have no effect on the beta- and gamma-range CMC during isometric compensation for modulated forces at 8% MVC. In addition, they support that the frequency range of CMC depends on a multiplicity of factors, *i.e*. task parameters, inter-individual differences and possibly the behavioral strategy applied by each individual.

## Methods

### Subjects

Eight healthy right-handed subjects (female, mean age 28 ± 10 years) without any history of neurological disease participated in the study. Handedness was tested according to the Oldfield questionnaire [27]. Three of the subjects had already participated in similar experiments in our lab. All subjects participated according to the declaration of Helsinki, with informed consent and the approval of the local ethics committee.

### Paradigm

**Figure 1:**
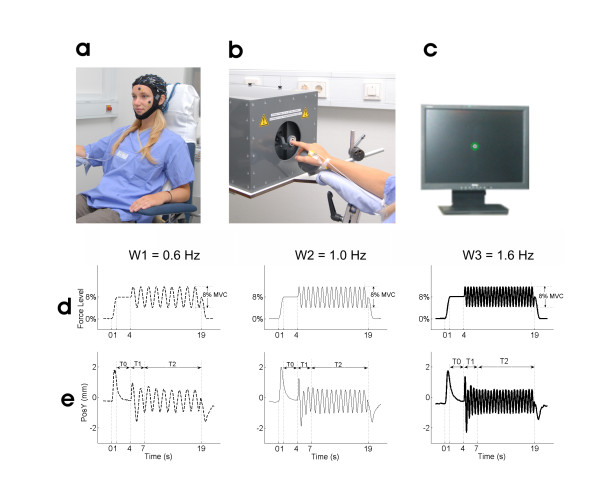
**Experimental apparatus and conditions**. (A) High-resolution EEG recorded from 58 scalp positions together with electrooculogram (EOG). (B) Home-made manipulandum and EMG recorded from FDI muscle during the experiment. (C) Visual feedback of the ring position displayed on a monitor in front of the subject. (D) Target force profiles in the three conditions W1 = 0.6 Hz, W2 = 1 Hz and W3 = 1.6 Hz. The force level was 8% MVC in all three conditions. (E) Grand average across subjects of finger position for the three conditions. Note the three time periods T0 (3 s static force period), T1 (3 s period of transient fluctuations), and period T2 (period with steady-state force compensation).

During the experimental session, the subject sat in an electrically shielded, dimly lit room. The right arm was supported by a splint and the subject was instructed to place the hand over a sphere and the right index finger in the ring of a home-made manipulandum (*see *Figure 1B).

The manipulandum was designed for applying vertical forces on the finger at the level of the metacarpophalangeal joint. A computer-controlled tooth belt drive produced a variable force on the ring. The subject had to compensate the force generated by the manipulandum isometrically to maintain the ring in its initial position (*see *Figure 1B). Visual feedback (*see *Figure 1C) about the position of the ring was provided via a 19'' monitor placed 100 cm in front of the subject and displaying two concentric circles. The green outer circle was located in the centre of the screen and represented the ring's reference position while the white inner circle moved corresponding to the ring's actual position. The subject had to maintain the small white circle inside the green one, so that when a given force was applied to the ring the subject had to compensate it by generating force in the opposite direction (here flexion). The sensitivity of the visual feedback with respect to the finger position corresponded to 2 mm on the screen for 1 mm ring displacement.

#### Force profile

The target force had four different phases (Figure 1D): a *ramp phase *(rising cosine function) which ensures a smooth start of the generated force. In all experimental conditions, the force level, or ramp amplitude, was 8% MVC. The 1 s ramp phase was followed by a 3 s-period of *static force *(T0) that gave time to stabilize the force to the "0" position (8% MVC). After the static period, the sinusoidally *modulated force *period with 8% MVC peak-to-peak amplitude and lasting 15 s was followed by *downward ramp phase *(again cosine function) to ensure a smooth end of the generated force.

#### Experimental conditions

Three different experimental conditions were investigated in a given recording session (Figure 1D):

• *0.6 Hz condition (W1): *The frequency of the force modulation was 0.6 Hz (Figure 1D, left panel).

• *1 Hz condition (W2): *the frequency of the force modulation was 1 Hz (Figure 1D, middle panel).

• *1.6 Hz condition (W3): *The frequency of the force modulation was 1.6 Hz (Figure 1D, right panel).

The three frequencies W1, W2, and W3 were carefully selected so that they were equidistant on a logarithmic scale, holding the following relations: $W2=\frac{5}{3}\times W1$ and, $W3=\frac{8}{5}\times W2$ where $\left(\frac{5}{3}=1.67\right)\approx \left(\frac{8}{5}=1.6\right)$. Therefore, W2 is approximately the geometric mean of W1 and W3. This selection is in line with the notion that signal discrimination in humans is usually following logarithmic rules (see [28]). Besides, any single stimulus frequency would not overlap with the frequency spectrum of another stimulus frequency including its harmonics in order to reduce unwanted crosstalk [29].

Prior to the experiment, we recorded rest EEG for 5 minutes while subjects were attending at the small white circle and their right hand was resting over the sphere with the right index finger in the ring of the manipulandum. During this rest period no force was applied by the manipulandum, so that the index finger remained static in its initial position. After that the force corresponding to the individual MVC was measured. An experimental session consisted of 5 different recording series, while each series included 18 trials. The 3 experimental conditions W1, W2 and W3 were presented in a pseudo-randomized fashion within the 18 trials, so that each frequency appeared 6 times within one recording series. Therefore, a total of 30 trials were recorded in each subject for each of the three frequency conditions. To avoid muscle fatigue, rest intervals of 7 to 12 s were included between the trials and approx. 5 min between the series.

To optimize performance and to avoid attentional variation across trials, the subjects were requested to concentrate on the temporal structure of the applied force profile and to tune the isometric contraction of their finger muscles to the identified force frequency. After each trial, they had to verbally report the frequency of the force modulation as 'slow', 'middle' and 'fast', corresponding to the frequencies 0.6 Hz (W1), 1 Hz (W2), and 1.6 Hz (W3) respectively.

The subjects were instructed to avoid any other movements and to fix their gaze on the concentric circles displayed on the screen. Before the onset of the recordings, subjects performed some trials to get familiarized with the task.

### Recordings

Electrical potentials (bandpass 0-200 Hz, sampling rate 1000 Hz) were recorded from 58 scalp positions according to the international 10-10 system (Synamp 2, NeuroScan, El Paso, TX, USA) referenced to Cz (Figure 1A) with ground at FzA. Electrode impedances were kept under 5 kOhm. The electrooculogram (EOG, same bandpass and sampling rate as for EEG) was recorded to exclude trials contaminated with eye movements from further analysis. Electromyographic activity (EMG, bandpass 0-200 Hz; sampling rate 1000 Hz) was recorded with surface electrodes using a belly-tendon montage from three muscles: the pars indicis of the right flexor digitorum superficialis (FLE), prime mover of the index finger flexion, the right first dorsal interosseus (FDI), and the right extensor digitorum communis (EXT).

### Data analysis

#### Performance

In each condition the recorded finger position was first cut into 30 epochs of 24 s each starting from 2 s before the onset of the trial (Figure 1E). Then for each participant, the temporal profile of the mean finger position was obtained by averaging the 30 epochs, and the grand average of the finger position was computed across all participants. Based on the grand average of the finger position, three periods (T0, T1 and T2) were identified (Figure 1E). The 3 s static force period was named T0. T1 corresponded to the period during which the finger position showed large transient fluctuations. T2 corresponded to the period in which the finger position reached a steady state and remained stable until the end of the force modulation.

#### EEG-EMG coherence analysis

Only data from period T1 and T2 was included in the analysis. In each trial, the data recorded during the 15 s sinusoidal force modulation (Figure 1E) was first separated in two data sets corresponding to periods T1 (3 s) and T2 (12 s). Then, for both periods (T1 and T2), data was further cut into segments with an overlap of 50%. Segments had duration of 1 s, therefore allowing a frequency resolution of 1 Hz for further spectral analysis. Artifact rejection was visually performed off-line trial-by-trial to exclude segments contaminated with eye movements. The EEG signal was then transformed into the reference-free current source density distribution (CSD) which reflects the underlying cortical activity [30]. The CSD algorithm was estimated using the spherical spline interpolation method [31] implemented in the commercial software 'BrainVision' 1.05 (München, Germany). For each subject, 100 artifact-free segments were obtained from the period T1, while 400 segments were obtained for the period T2.

EMG signals were rectified, as it is known that full-wave rectification, providing the temporal pattern of grouped firing motor units [32], is an appropriate procedure for power and coherence analysis [33]. The discrete 1000 points Fourier transform was computed for each segment.

#### Calculation of the EEG spectral power (SP) and the EEG-EMG coherence (CMC)

Power spectrum (SP) for a given channel *(c) *was further calculated according to the following equation

(1) $$SP_{c}\left(f\right)=\frac{1}{n}\sum_{i=1}^{n}C_{i}\left(f\right)C_{i}^{*}\left(f\right)$$

where *C_i _*represents the Fourier transformed channel *c *for a given segment number *(i = 1....n) *and '*' indicates the complex conjugate.

*Coherence values *were calculated between the rectified EMG and the EEG channels overlying the sensorimotor area contralateral to the active hand (SM1c) in order to calculate the synchronization between the two signals. Coherence values were calculated on the basis of the following formulae:

(2) $$Coh_{c1,c2}\left(f\right)=\frac{{\left|S_{c1,c2}\left(f\right)\right|}^{2}}{\left|SP_{c1}\left(f\right)\right|\times \left|SP_{c2}\left(f\right)\right|}$$

where

(3) $$S_{c1,c2}\left(f\right)=\frac{1}{n}\sum_{i=1}^{n}C1_{i}\left(f\right)C2_{i}^{*}\left(f\right)$$

thus *Sc_1_,c_2_(f) *is the cross-spectrum for the EEG signal channel *c1 *and the rectified EMG signal in channel *c2 *at a given frequency *f *and *SPc_1_(f) *and *SPc_2_(f) *are the respective power spectra for *c1 *and *c2 *at the same frequency. For frequency *f*, the coherence value, *Coh(c_1_,c_2_)(f)*, thus corresponds to the squared magnitude of a complex correlation coefficient. *Coh(c_1_,c_2_)(f) *is then a real number between 0 and 1.

As coherence was estimated based on overlapped segmentation, CMC is considered to be significant if the resulting value lies above the confidence level *(CL) *according to a recent developed method for coherence estimation [34]:

(4) $$CL(\alpha )=1-{\left(1-\alpha \right)}^{\frac{1}{L-1}}$$

where 2*L *is termed as the number of degrees of freedom of a coherence estimate. For the calculation of L, we refer the reader to the original paper [34], where the method is explained in full detail. Alpha, '*α*', is the desired level of confidence. We considered coherence to be significant over the upper 95% confidence limit.

We focused on the strongest coherences that were obtained between the EEG channels (C1 or C3) over the left sensorimotor area contralateral to the right index finger movement and the rectified EMG. This procedure may account for the different location of the maximum CMC peak (C1 or C3) due to inter-individual differences in brain morphology.

#### Analysis of CoG (Center of Gravity)

To detect frequency shifts within the coherence spectrum for the beta and gamma-range, we also calculated the centre of gravity (CoG), *i.e*. the frequency at which all CMC activity within the beta- and gamma-range coherence could in theory be concentrated; around this frequency point, the CMC is balanced. This was done according to:

(5) $$CoG=\frac{\sum_{s=1}^{n}f_{s}\times C_{s}}{\sum_{s=1}^{n}C_{s}}$$

where s = 1 ... n indicates the number of significant bins with its respective frequency value f and coherence amplitude C.

#### Analysis of position error

To estimate a possible relationship between CMC, spectral power and performance, we made an analysis of the position error (PE) within the period T2. Similarly as for the analysis of EEG-EMG coherence, 100 and 400 segments of 1 s length were obtained from finger position signal in the periods T1 and T2 respectively. Then, we calculated the mean of the position error magnitude (|*E_k,i_*|) within each 1s segment *i*. A global measure PE was obtained by computing the mean of position error over all segments *n *on the basis of the following formulae:

(6) $$PE=\frac{1}{s.n}\sum_{i=1}^{n}\left(\sum_{k=1}^{s}\left|E_{k,i}\right|\right)$$

where *k *= 1...s is the sampled point in the actual segment *i*, *i *= 1...n is the segment number (100 for T1 and 400 for T2) and s = 1000 is the number of sampled points in each segment.

#### Statistical analysis

To test for any statistical difference in CMC and cortical motor spectral power (SP) between the three frequencies of force modulation, we first measured the area under the coherence curve and above the significance level, *A_coh_*, and under the spectral power curve, *A_pow_*, in-between two frequency windows: 15-32 Hz for the beta-range and 25-45 Hz for the gamma-range. The partitioning into beta- and gamma-range used in the present study does reflect current thinking with respect to neural systems and especially in the motor system [18].

Individual values for the values *A_coh _*for CMC and position error (PE) were first transformed logarithmically to yield symmetric distributions according to the formulae:

(7) $$A{'}_{coh}=\log\left(0.02+A_{coh}\right)-\log\left(0.02\right)$$

(8) PE'=log(0.4+PE)−log(0.4)

Afterwards repeated measures ANOVA with three factors *subject *(beta, gamma), *period *(T1, T2), and *frequency *(W1, W2, W3) were applied to the values *A'_coh_*, *A_pow _*for SP, CoG, and PE. All interactions were assessed.

To check for linear relationship between the *PE *values and the frequencies (W1, W2, W3), we applied repeated ANOVAs including an analysis of the polynomial contrast (linear and quadratic).

#### Detection rate

For each experimental condition, "detection rate" (DR) was computed for each subject in each condition to evaluate the accuracy with which participants identified the frequency of the modulated force. DR was defined as the percentage of trials (from a total of 30) where subjects correctly identified the frequency of the force modulation. The Friedman test was applied to compare values of detection rate (DR) for the three frequency conditions for each single subject, with the null hypotheses that the distributions of the values tested are the same across all three conditions.

## Results

### Ramp phase (period T0)

Figure 1D shows the target force profiles for the three conditions W1, W2, and W3. In all three conditions fluctuations (~ 3 mm) of the ring position during T0, *i.e*. the static force following the force ramp, were observed (Figure 1E). The force ramp was compensated by all subjects before the end of the period T0, so that the finger came back to the '0' position before the onset of the force modulation.

### Dynamic modulated force period (periods T1 and T2)

For W1, W2 and W3 the grand-averages of the finger position during the 30 trials in all participants are shown in Figure 1E. The frequency of the oscillations corresponded to the target frequency in all three conditions. Note that finger oscillations started with larger amplitudes during the initial 3 s (period T1) but stabilized at slightly lower amplitudes during the period T2, where a steady-state oscillatory performance is observed around the '0' position with deviations of maximally 1.5 mm in both directions.

### Detection rate (DR)

**Figure 2:**
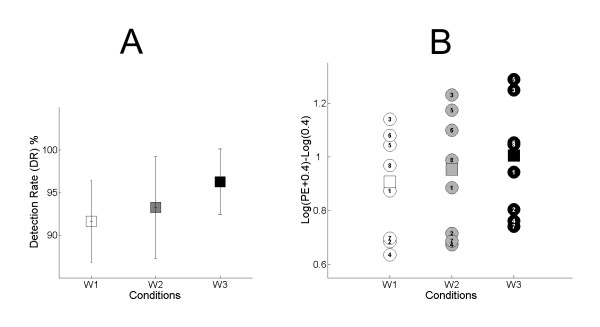
**Behavioral performance**. (A): Mean values and standard deviation of Detection Rate (DR) for all 8 subjects for conditions W1, W2 and W3. (B): PE values from each individual subject are represented as circles. Squares represent the mean values of Position Error (PE) in the period T2 for condition W1 (white), W2 (grey) and W3 (black). Note that PE increases linearly from W1 to W2 to W3.

Figure 2A shows the mean DR values for the three conditions W1, W2 and W3 for all subjects. One can see that subjects successfully identified the frequency of the force (as slow, middle or fast) in more than 90% of the 30 trials. In addition, the DR showed a tendency to increase from ~90% to ~95% with higher frequency of force modulation, so that the highest detection rate (DR) was observed for W3. This tendency was present in most of the subjects. This suggests that subjects were more aware of the temporal force profile when the force modulation rate increased. However, the Friedman test for the DR values did not show any significant difference among the three conditions.

### Position error (PE) for periods T1 and T2

The statistical comparison of the performance errors (PE) in the two periods T1 and T2 revealed a highly significant difference (F(1,7) = 95, p < 0.001), reinforcing the fact that fluctuations in finger position were higher during the transition period T1, as can be seen in the grand-average of the finger position (Figure 1E). In addition, PE significantly increased at higher frequencies (F(1,7) = 10.7, p < 0.01, see Figure 2B). The analysis of the polynomial contrast showed that the increase of PE for higher frequencies was due to a linear effect (p = 0.02), the quadratic part being not significant.

### Beta- and gamma-range corticomuscular coherence (CMC)

**Figure 3:**
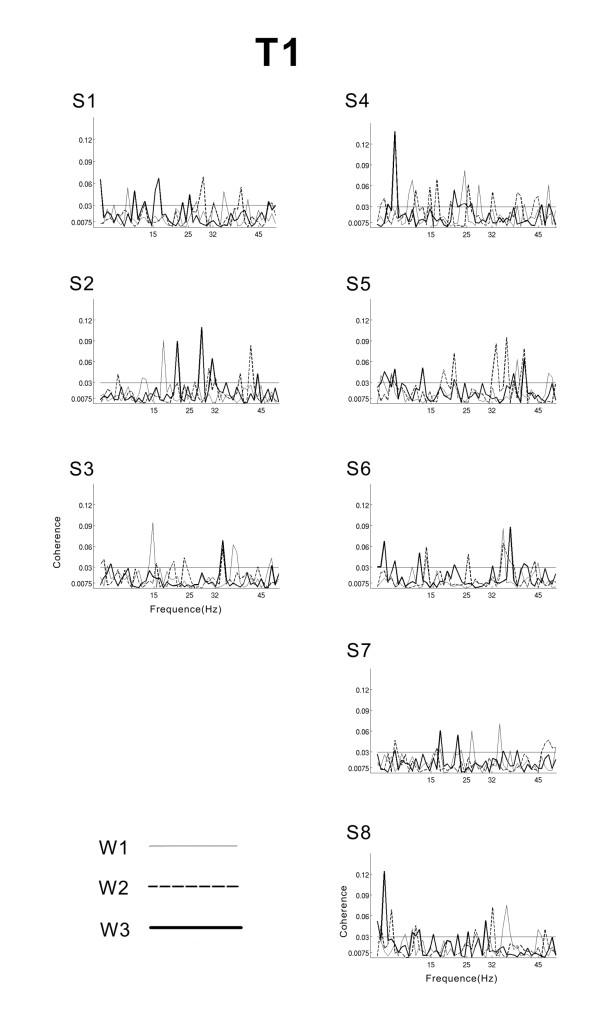
**Coherence spectra for the period T1**. Superimposed frequency-coherence plots for each subject in the beta- and gamma-range for the three conditions W1 (dotted line), W2 (thin line) and W3 (thick line) during the transitory period T1. The confidence level at 95% is marked with a horizontal thin line.

**Figure 4:**
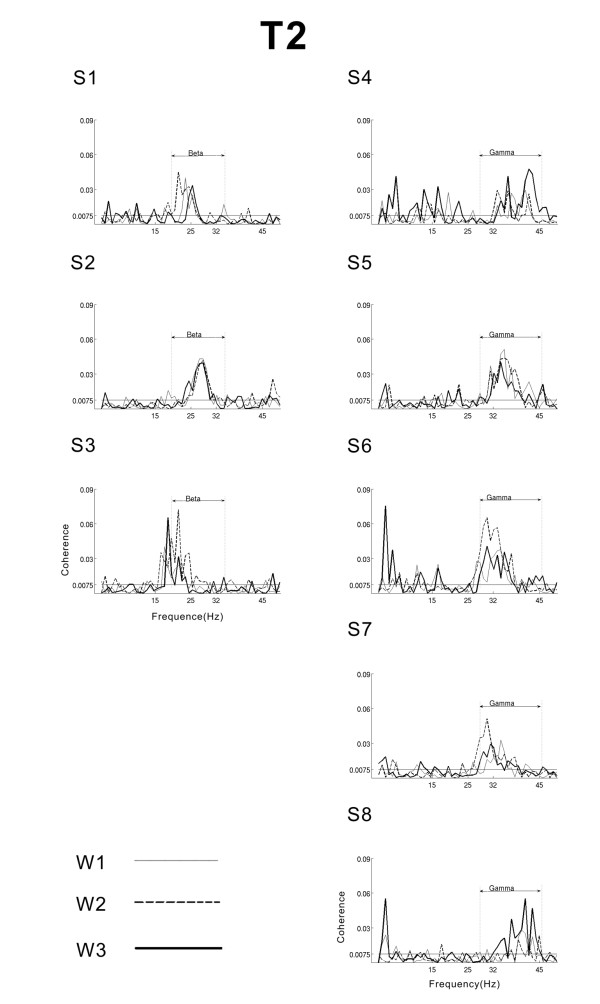
**Coherence spectra for the period T2**. Superimposed frequency-coherence plots for each subject in beta- and gamma-range for the three conditions W1 (dotted line), W2 (thin line) and W3 (thick line) during the period T2. The confidence level at 95% is marked with a horizontal thin line. Significant broad-band CMC are observed in period T2: with CMC in beta-range CMC in three subjects (left column) and in gamma-range in five subjects (right column). Note the presence of a narrow-band CMC peaking at around 2 Hz in all 5 gamma-subjects during 1.6 Hz force modulation.

The maximum EEG-EMG coherences were observed over the contralateral sensorimotor cortex (C3 or C1). Individual EEG-EMG coherence curves of the eight subjects for the 0.6 Hz, 1 Hz and 1.6 Hz conditions during the periods T1 and T2 are shown in Figure 3 and Figure 4 respectively.

During the transitory period T1 the CMC spectra contained several random-like sharp peaks distributed in the 15-45 Hz range (Figure 3).

In period T2, when motor performance reached a rather stable state, the CMC spectra showed consistent broad-band coherences within the beta- (15-32) and gamma (25-45 Hz) range (Figure 4). The three-way ANOVA revealed significant main effect of the factor *Period *with significantly higher CMC in T2 (F(1,7) = 21, p < 0.001).

As seen in Figure 4, five of the subjects have broad-band CMC mostly in gamma-range (25-45 Hz) while three subjects had it in beta-range (15-32 Hz), as supported by a significant main effect of factor *Subject *for the CoG (F(1,7) = 9, p < 0.01). The subjects were accordingly classified in more beta- and more gamma-group. Note that although the CMC data fits fairly well to this clear-cut classification, there are subjects with peaks in both beta and gamma-range. For example, in Figure 4 the gamma-subjects S4, S5 and S6 show lower peaks in beta-range. The classification in beta- and gamma-groups was meant to capture the major differences of the CMC pattern.

No significant effect of the main factor *Frequency *was found. Thus, the outcome of the statistical analysis reveals that neither the CMC amplitude nor the CoGs are influenced by the frequency of the modulated force.

### Low-frequency corticomuscular coherence (CMC) at the frequency of the force modulation

We also looked for significant CMC values within the 0 - 5 Hz range, peaking at the frequencies of force modulation (W1, W2 or W3) or their harmonics.

All five gamma subjects had a low-frequency CMC peak at 2 Hz, that matches the frequency modulation W3 (1.6 Hz), according to the 1 Hz spectral resolution. This low-frequency CMC peak was consistently observed in the 1.6 Hz condition only (Figure 4).

**Figure 5:**
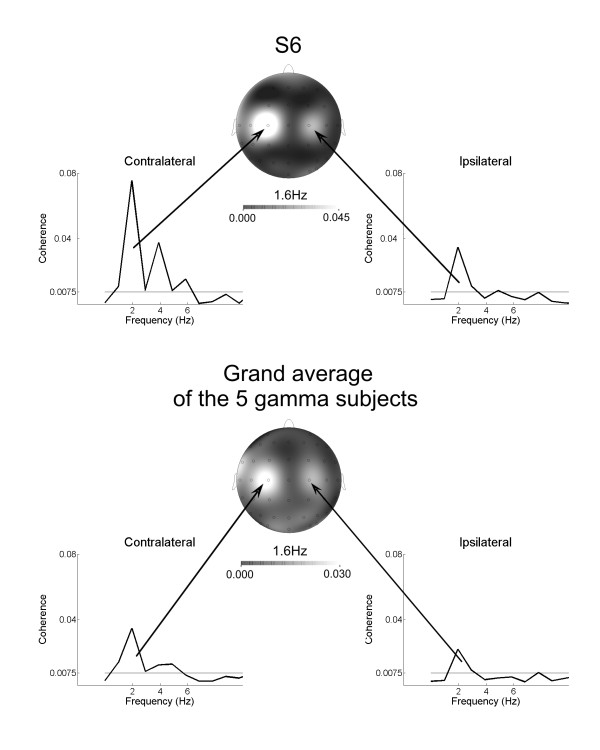
**Topographic distribution of the low-frequency CMC peak for condition W3**. Frequency-coherence plots in the range 1-10 Hz and topographic distribution of CMC for subject S6 (upper row) and grand-average of all five gamma-range subjects (lower row) for condition W3. Note the bilateral organization of the corticospinal coherence, with stronger activation over the contralateral sensorimotor area.

This CMC peak at 2 Hz in W3 condition was observed not only over the contralateral, but also over the ipsilateral sensorimotor cortex, as displayed in the topographic maps of one individual and of the grand average in Figure 5. Significant low-frequency CMC peaks at 2 Hz were observed only in electrodes above sensorimotor areas.

None of the three beta-group subjects had such a peak (see Figure 4, left column).

### Cortical motor spectral power (SP)

With respect to the cortical motor SP amplitude, the ANOVA did not reveal any significant main effect for the three tested factors or their interactions. Subjects showing increased CMC in beta- or gamma-range did not show any corresponding increase in spectral power in the same frequency range.

### Cortical motor spectral power during rest

The cortical spectral power during rest did not show any differences between beta- and gamma-range subjects in terms of beta and gamma power.

## Discussion

The present study was designed to investigate the corticospinal interactions during isometric compensation for force modulated at three different frequencies (0.6, 1.0, 1.6 Hz). A stable CMC and performance only occurred after a transitory phase in which the force had to be adjusted to the modulation frequency. During the transitory phase (period T1), the CMC spectra contained several random-like sharp peaks distributed in the 15-45 Hz range. This pattern may arise due to a transitory regime of corticospinal circuits functioning during the first 3 s. During the stationary phase (Period T2), CMC occurred in five of the eighth subjects in gamma-range and in three of them in beta-range. The findings from the study show the presence of significant broad-band gamma-range CMC in five of the eight subjects and beta-range in three of them. Neither the gamma-range CMC, nor the beta-range CMC were modified by the various force modulation frequencies. In addition, a sharp CMC peak at 2 Hz was observed only during the highest frequency of force modulation (1.6 Hz) suggesting that corticospinal circuits resonating at the force frequency also play an important role in our isometric force compensation task.

### Beta- and gamma-range CMC are not modified by the frequency of force modulation

We were specifically interested in studying CMC during high-precision slow-paced natural movements which typically occur within the 0-2 Hz frequency range. The findings show that increasing the force modulation frequency within this range did not induce any changes in amplitude or frequency range of the beta- and gamma-range CMC.

Any interpretation of this result should take into consideration the following: Corticospinal oscillations may be actually modulated by the frequency of the force, but this effect could not be manifested in our experimental design due to the specific frequency range of interest (0.6 - 1.6 Hz). This range was delimited by several constraints: *First*, the upper extreme of this range was set at 1.6 Hz to avoid muscular stiffness associated to higher frequencies, while the lower extreme was set above 0.5 Hz to facilitate the sensory perception of the dynamically modulated force. *Second*, CMC could be actually modulated by the force frequency even within this range of interest, but we cannot easily detect this effect with non-invasive recordings such as EEG or MEG due to spatiotemporal smearing of neighboring neural sources [35,36]. Therefore, intracortical recordings are needed to further clarify this issue. Within these constrains and limitations, our results rather align with the view that CMC is not modulated neither by the amplitude [23] nor the frequency of the modulated force.

### Both beta- and gamma-range CMC are associated to isometric compensation of modulated forces at 8% MVC

The finding of five gamma- and three beta-range subjects is difficult to interpret when considering that performance error (*PE*) and awareness of the modulation frequencies as reflected in the *DR *did not differ significantly between the two groups. Thus, difference in the CMC frequency range was not correlated to behavioral performance. Moreover, this finding was not related to differences in experience in similar experiments. These results suggest that there are intrinsic inter-individual differences in CMC, whereas subjects may equally perform the visuomotor task recruiting different functional corticospinal circuits resonating in the beta- or gamma range. We described this in a previous work of ours investigating the CMC during modulated forces at 8% MVC [17].

However, in addition to the interindividual differences some other differences could be taken into consideration: The beta- and gamma-subjects may have differed in their strategy to compensate for the modulated forces. Subjects were requested to attend to the force modulations in order to recognize and report the frequency as slow, middle or fast. Nevertheless, subjects may have chosen: ***i) ***to encode precisely the temporal profile of the force and reproduce the "dynamic" pattern, generating a force in the opposite direction, or ***ii) ***to exert a "pseudo-static force" at the 8% MVC level, independently of the modulation frequency. The first, "dynamic", strategy may elicit corticospinal oscillations in gamma-range to rapidly and continuously integrate proprioceptive, visual, and cognitive (preparatory attention) information and meet the demands of a dynamic environment [18]. The second, "pseudo-static", strategy, even in the presence of a dynamic modulated force, is mainly relying on the static features of the force profile (the 8% MVC force level) and may lead to beta-range CMC, which is shown to be associated with rather rigid and stable regimes of corticospinal interactions [11,37,38]. Although not supported by differences in behavioral performance it could be speculated that the five gamma subjects were applying a "dynamic" strategy, while the three beta subjects, although aware of the force temporal profiles, performed the task in a "pseudo-static" way.

Our previous studies reported beta-range CMC during isometric compensation for low-level static forces at 4% MVC and predominantly gamma-range (30-45 Hz) CMC during dynamic force compensation [8,11,18,23]. However, in a recent investigation of the CMC during a visuomotor task where different levels of a modulated force (8, 16, 24% MVC) were applied [17], we also found broad-band beta-range CMC. Taken together, we conclude that beta-range CMC is not confined to or specific for low-level static forces only. Rather, the sensorimotor system may resort to either beta- or gamma-range CMC to generate effective corticospinal interaction when compensating for dynamic modulated forces. Both beta- and gamma-range CMC represent mechanisms for effective corticospinal interaction and can be selectively used to subserve different functions [8,15].

In her review article Tallon-Baudry [39] concludes that there is no one-to-one relationship between a frequency band and a single cognitive function. Our results demonstrate that there is no one-to-one relationship between frequency band and specific motor function either.

### The bilateral low-frequency CMC peak in the gamma subjects may fit increasing demands at higher frequency of force modulation

For the highest force modulation frequency investigated (1.6 Hz) we found an additional sharp CMC peak at the same frequency. This low-frequency peak was observed only in the five gamma subjects. Similar CMC patterns over the sensorimotor area have been observed in recent studies of corticospinal interaction for tasks requiring to synchronize rhythmic foot movements or periodic isometric contraction of calf muscles to external periodic events [40]. In this work, a CMC peak at the frequency of the movements or muscle contraction was associated with corticospinal synchronization processes during dynamic motor output. Following the same line of reasoning, we interpret the co-existence of gamma-range CMC and of a CMC peak at 1.6 Hz (modulated force frequency) as support to our view of gamma-range CMC as reflecting effective corticospinal interaction. The fact that this CMC peak at 2 Hz was found only for the highest frequency tested (1.6 Hz) can be explained when considering that in this condition the performance errors were significantly larger, indicating an increase in task difficulty. In fact, previous work by Flowers [41] has shown that difficulties in performance of a tracking task increase for frequencies above 1.5 Hz. Therefore, this low-frequency CMC may be related to the increasing task demands. The bilateral sensorimotor organization of the CMC peak at 2 Hz (see Figure 5) supports this suggestion as it has been shown that increasing motor task complexity leads to the recruitment of larger neural resources and an enhanced functional cooperation between both contralateral and ipsilateral motor areas [42-44].

Nesting of high-frequency oscillations (gamma) into low-frequency ones (theta) is suggested to multiplex processes in the same location [39,45,46]. Lakatos et al. [47] hypothesized that slower rhythms provide windows, in which the high-frequency rhythms are activated. It is possible that in our study the nesting of the gamma-range CMC into 1.6 Hz oscillations contributes to cope with the higher task demands in this condition.

The detection rate (DR) was higher for higher force frequencies, with value above 95% for the 1.6 Hz modulation. This result is in line with studies showing that perceptual awareness of external periodic events increase with the rate of change of these events [48,49]. However, a relationship between awareness of force frequency and a CMC peak at the same frequency remains at best highly speculative, and should be addressed in experimental paradigms where level of awareness of the force profiles are explicitly manipulated.

## Conclusions

Our findings suggest that the frequency of force modulation has no effect on the beta- and gamma-range CMC during isometric compensation for modulated forces at 8% MVC. The beta- and gamma-range CMC may be related to interindividual differences. The sharp CMC peak at 2 Hz during the highest frequency of force modulation (1.6 Hz) suggest that corticospinal circuits resonating at the force frequency also play an important role in isometric force compensation. Our results are a step towards further understanding of the global oscillatory processes and help to get new insights in the dynamics of neural systems [50].

## List of Abbreviations

CMC: Corticomuscular Coherence; EEG: Electroencephalography; EMG: Electromyography; PE: Position Error; DR: Detection Rate

## Authors' contributions

JRN, RK, CM and FH designed the experiment. XW and JRN ran the experiments. XW, JRN and JSM performed the quantitative analyzes of the data. JRN, RK, and MCHR wrote the manuscript. RK conceived the study and participated in all the steps of the realization of the study. All the authors read and approved the MS and its revision. Statement of consent was given for publication of the images.
